# Initial Experience With the Trevo NXT Stent Retriever

**DOI:** 10.3389/fneur.2021.704329

**Published:** 2021-07-16

**Authors:** Manina M. Etter, Markus Möhlenbruch, Charlotte S. Weyland, Carlos Pérez-García, Manuel Moreu, Francesco Capasso, Nicola Limbucci, Omid Nikoubashman, Martin Wiesmann, Kristine Blackham, Ioannis Tsogkas, Peter Sporns, Johanna Maria Ospel, Alex Brehm, Marios-Nikos Psychogios

**Affiliations:** ^1^Department of Neuroradiology, Clinic for Radiology and Nuclear Medicine, University Hospital Basel, Basel, Switzerland; ^2^Department of Neuroradiology, University Hospital Heidelberg, Heidelberg, Germany; ^3^Department of Neuroradiology, Hospital Clínico San Carlos, Madrid, Spain; ^4^Neurovascular Interventional Unit, Careggi University Hospital, Florence, Italy; ^5^Department of Neuroradiology, University Hospital RWTH Aachen, Aachen, Germany

**Keywords:** ischemic stroke, mechanical thrombectomy, stent retriever, primary combined approaches, medium vessel occlusion, large vessel occlusion

## Abstract

**Background:** The application of a new coating to the delivery wire of the Trevo retriever has the potential to improve its handling. We therefore report our initial experience with this new stent retriever for mechanical thrombectomy of large and medium vessel occlusions.

**Methods:** We pooled data of four high-volume European stroke centers over the time period from October 2020 to February 2021. Patients were included in our study if the Trevo NXT stent retriever was used as a first-line device. Primary endpoints were first-pass near-complete or complete reperfusion, defined as mTICI score of ≥2c. Secondary endpoints were final reperfusion, National Institutes of Health Stroke Scale (NIHSS) at 24 h and discharge, device malfunctions, complications during the procedure, and subjective ratings of the interventionalists regarding device functionality.

**Results:** Eighty patients (39 women, mean age 74 ± 14 years) were eligible for our study. Median NIHSS at admission was 15 (IQR, 8–19), and median Alberta Stroke Program Early CT Score at baseline was 9 (IQR, 8–10). In 74 (93%) patients a primary combined approach was used as first-line technique. First-pass near-complete reperfusion was achieved in 43 (54%) and first-pass complete reperfusion in 34 (43%) patients. Final near-complete reperfusion was achieved in 66 (83%) patients after a median of 1.5 (1–3) passes, while final successful reperfusion was observed in 96% of our cases. We observed no device malfunctions. Median NIHSS at discharge was 2 (IQR, 0–5), and 3 patients (4%) suffered a symptomatic intracranial hemorrhage.

**Conclusions:** Based on our initial data, we conclude that the Trevo NXT is an effective and safe tool for mechanical thrombectomy especially when used for combined approaches.

## Introduction

Mechanical thrombectomy is considered the standard of care for ischemic strokes caused by large vessel occlusions (LVO) (1). The main techniques can be generally divided into three major categories: (I) use of a stent retriever (SR) and subsequent withdrawal of the device with or without flow arrest by a balloon-guide catheter (BGC); (II) direct aspiration technique using a large-bore aspiration-catheter (ADAPT), placed at the face of the clot; and (III) primary combined approaches (PCA), where both an SR and an aspiration-catheter are used intracranially with additional extracranial aspiration through the guide catheter (2–4).

Among the different techniques for mechanical thrombectomy, various SRs are used in combined approaches. The Trevo ProVue SR is a well-established mechanical thrombectomy device as several studies have confirmed its efficacy in endovascular stroke therapy. In the Multicenter Randomized CLinical trial of Endovascular treatment for Acute ischemic stroke in the Netherlands (MR CLEAN), Trevo was the most frequently used SR (5). In the Clinical Mismatch in the Triage of Wake Up and Late Presenting Strokes Undergoing Neurointervention With Trevo (DAWN) randomized trial, which showed thrombectomy to be superior compared to medical management alone, Trevo was the only SR used in the interventional arm (6). The device was also assessed in the Trevo retriever Registry, a prospective post-market study which documented reperfusion success after mechanical thrombectomy and functional outcomes at 90 days. Reperfusion success was defined by a modified thrombolysis in cerebral infarction (mTICI) score ≥2b, which was reached in 93% of the study population. Furthermore, functional independence at 90 days (defined as a modified Rankin Scale ≤2) was documented in 55% of patients (7).

In this retrospective study, we assessed the new-generation Trevo SR (Trevo NXT) for endovascular therapy of ischemic stroke due to large or medium vessel occlusions. We focused on its efficacy and peri-interventional safety.

## Materials and Methods

### Study Design and Patient Population

We pooled data of four high-volume European stroke centers over the time period from October 2020 to February 2021. Patients were included in our study if the Trevo NXT ProVue SR (Stryker Neurovascular, Kalamazoo, MI, USA) was used as the first-line device. No other inclusion or exclusion criteria were applied.

The National Institutes of Health Stroke Scale (NIHSS) was obtained at baseline, 24 h post procedure and at discharge by a certified stroke neurologist. All angiographies were rated by the treating interventionalist using the mTICI (8) scale. Non-contrast computed tomography (CT) or magnetic resonance imaging (MRI) was regularly performed within 24 h after treatment or immediately in symptomatic patients. Symptomatic intracranial hemorrhage (sICH) was defined as an intracranial hemorrhage that was associated with clinical deterioration, as documented by an increase of ≥4 points on the NIHSS. In intubated patients, sICH was defined by the European Cooperative Acute Stroke Study-2 (ECASS-2) criteria as any parenchymal hematoma grade I or larger (9).

As the company claims that the new Trevo NXT can be delivered and retracted through the microcatheter or the aspiration-catheter more easily compared to earlier Trevo generations, we surveyed the involved interventionalists on their subjective experience with the new device. The following questions were used: (a) How easy was advancing the SR through the microcatheter? (b) Could target placement of the SR be achieved? (c) How easy was retrieving the SR into the aspiration-catheter/BGC? and (d) In PCA, could a stable wedge position be achieved, or was the SR accidentally withdrawn into the aspiration-catheter? For questions (a) and (c) we used an evenly distributed ordinal five-point scale (ranging from 1 = very easy, 2 = easy, over 3 = neutral, to 4 = hard, and 5 = very hard), while (b) and (d) were yes/no questions. For this analysis, only cases in which a PCA was used as the first-line approach were included.

The primary endpoint was first-pass complete or near-complete reperfusion, defined as an mTICI ≥2c. Secondary endpoints were final reperfusion, NIHSS at 24 h and discharge, occurrence of sICH, device malfunctions, complications during the procedure, and the subjective ratings of the interventionalists.

### Endovascular Procedure

Procedures were performed under conscious sedation, local anesthesia, or general anesthesia. Vital findings were monitored by anesthesiologists or stroke neurologists in all patients during the procedures. All procedures were done using a femoral artery access. The treating physician was free to choose the first-line technique, but in most cases the Stent retriever Assisted Vacuum Extraction (SAVE) technique was used as described elsewhere (4).

### Device Characteristics

The Trevo NXT ProVue retriever is a further development of its predecessor, the Trevo XP ProVue retriever. While the stent itself was not changed and still offers full radiopaque visibility, the delivery wire received a new hydrophilic coated polymer jacket which enables a smoother and easier delivery and improved retraction into the aspiration-catheter. It is delivered through a 0.021-inch microcatheter (the 3 × 25 mm retriever can also be delivered through a 0.017-inch microcatheter). The device is available in working lengths from 25 to 35 mm and diameters from 3 to 6 mm. The wire length was increased to 200 cm, which improves its compatibility with tri-axial setups.

### Statistical Methods

Statistical analysis was performed using GraphPad Prism 9 (GraphPad Software, San Diego, CA, USA, https://www.graphpad.com/, 2021). Parametric variables are stated as mean ± standard deviation (SD). Non-parametric or ordinary variables are presented as median and interquartile range (IQR). No interference statistics were performed.

## Results

Out of 97 received data sheets, 80 patients were enrolled into this study. Ten of the 97 patients were excluded due to ADAPT being the first-line therapy, while in seven patients an SR other than the Trevo NXT ProVue was used for the first maneuver. All baseline characteristics are depicted in Table 1. Mean age was 74 ± 14 years, and 39 patients (49%) were female. The median NIHSS at admission was 15 (IQR, 8–19). Occlusion sites were internal carotid artery terminus (“ICA-T”) in 14 (18%), M1-segment in 38 (47%), M2-segment in 22 (28%), basilar artery in 4 (5%), P2-segment in 1 (1%), and vertebral artery in 1 (1%) patient. Median Alberta Stroke Program Early CT score (ASPECTS) on initial imaging was 9 (IQR 8–10). The majority of patients was treated either with a PCA (47/80) or PCA with balloon-guide (27/80), while the rest was treated with SR only (1/80) or with SR plus BGC (5/80). Most of the procedures were performed under conscious sedation or local anesthesia (67 patients). General anesthesia was used in the remaining 13 patients (16%).

**Table 1:** Baseline characteristics of the patients.

**Characteristics**	**Values**
No. of patients	80
Women, *n* (%)	39 (49%)
Age, mean ± SD	74 ± 14
NIHSS at admission, median (IQR)	15 (8–19)
Pre-stroke mRS, median (IQR)	0 (0–1)
ASPECTS at baseline, median (IQR)	9 (8–10)
**Primary approach**	
Stent retriever + BGC, *n* (%)	5 (6%)
PCA, *n* (%)	47 (59%)
PCA + BGC, *n* (%)	27 (34%)
Stent retriever only, *n* (%)	1 (1%)
**Occlusion location**	
ICA-T, *n* (%)	14 (18%)
M1, *n* (%)	38 (48%)
M2, *n* (%)	22 (28%)
BA, *n* (%)	4 (5%)
P2, *n* (%)	1 (1%)
VA, *n* (%)	1 (1%)
Tandem occlusion, *n* (%)	12 (15%)
**Outcomes**	
NIHSS at 24 h, median (IQR)	4.5 (1.5–11)
NIHSS at discharge, median (IQR)	2 (0–5)
sICH, *n* (%)	3 (4%)
Secondary distal emboli, *n* (%)	4 (5%)
Emboli to new territory, *n* (%)	1 (1%)

*ASPECTS, Alberta Stroke Program Early CT Score; BA, basilar artery; BGC, balloon-guide catheter; ICA-T, internal carotid artery terminus; IQR, interquartile range; NIHSS, National Institute of Health Stroke Scale; mRS, modified Rankin scale; PCA, primary combined approach; SD, standard deviation; sICH, symptomatic intracranial hemorrhage; VA, vertebral artery*.

First-pass complete or near-complete reperfusion (mTICI ≥2c) was achieved in 43 (54%) with complete reperfusion (mTICI 3) in 34 (43%) patients. The final complete or near-complete reperfusion (mTICI ≥2c) was observed in 66 (83%) and complete reperfusion (mTICI 3) in 45 (56%) patients after a median of 1.5 passes (IQR, 1–3). The rate of successful reperfusion (mTICI ≥2b) was 64% after one pass and 96% at the end of the procedure (Table 2). The overall median time from groin puncture to reperfusion was 39 min (IQR, 23–58.5). We documented 1 (1%) embolus in new territory (initial M1 to A1), and in 4 cases (5%) distal emboli were observed. In 3 patients (4%) an sICH was observed. In 2 cases the intracranial hemorrhage (ICH) was observed immediately on the post-mechanical thrombectomy scan in the angio-suite, while in the third case no immediate scan after MT was performed. It was observed on the first follow-up CT 4.5 h after the intervention. One ICH was mainly an extensive subarachnoid hemorrhage (SAH) in the basal cisterns and hemispheric sulci with small parenchymal hematoma in the left basal ganglia (affecting putamen) and insula; the other 2 ICHs were mainly parenchymal hematomas within the infarct area (both media territory left). In the case with the SAH no intravenous lysis was given, while in the other 2 cases it was given prior to the intervention. None of the sICHs were rated to be related to the intervention. In all cases the handling of the SR was continuously smooth. The median NIHSS 24 h post procedure was 4.5 (IQR, 1.5–11) and 2 (IQR, 0–5) at discharge.

**Table 2:** Angiographic results stratified by occluded vessel and overall vessels.

	**ICA-T**	**M1**	**M2**	**Overall**
No. of patients	14	38	22	80
**First-pass reperfusion**				
mTICI ≥2b, *n* (%)	5 (36%)	25 (66%)	11 (50%)	51 (64%)
mTICI ≥2c, *n* (%)	4 (29%)	20 (53%)	10 (46%)	43 (54%)
mTICI 3, *n* (%)	2 (14%)	15 (40%)	8 (36%)	34 (43%)
**Final reperfusion**				
mTICI ≥2b, *n* (%)	13 (93%)	36 (95%)	22 (100%)	77 (96%)
mTICI ≥2c, *n* (%)	12 (86%)	30 (79%)	19 (86%)	66 (83%)
mTICI 3, *n* (%)	7 (50%)	22 (58%)	13 (59%)	45 (56%)
No of passes, median (IQR)	3 (1–4)	1 (1–2)	1 (1–2)	1.5 (1–3)
Groin puncture–reperfusion time (min), median, IQR	51 (33.8–82.5)	34 (22.3–52.8)	35 (22–57)	39 (23–58.5)

*ICA-T, internal carotid artery terminus; IQR, interquartile range; mTICI, modified thrombolysis in cerebral infarction*.

A total of 158 passes were performed with the Trevo NXT; no device malfunctions were observed. Four complications were reported: 2 SAHs and 2 vasospasms (which resolved after application of nimodipine). Both vasospasms were observed in the segment where the SR was placed [one in the M2-segment of the right middle cerebral artery (MCA) and one in the M2-segment of the left MCA]. In both cases the Trevo NXT 4 ×35 mm SR was used.

A subgroup analysis of the angiographic results after stratification by occlusion site indicated higher first-pass complete or near-complete reperfusion results in the M1- and M2-segments compared to the ICA-T (53%/46% vs. 29%). Furthermore, the number of passes (median 3 for the ICA-T and 1 for the M1/M2) and the groin to reperfusion time (median 51 min for ICA-T vs. 34/35 min for M1/M2) were higher for ICA-T occlusions (see Table 2 for detailed results). The incremental improvement of the mTICI result declined from pass to pass (Table 3).

**Table 3:** Per pass reperfusion results.

**Pass #**	**1**	**2**	**3**	**4**	**5**	**6**
Number of patients	80	34	20	14	8	5
mTICI <2b, *n* (%)	29 (36%)	18 (53%)	12 (60%)	8 (54%)	5 (63%)	3 (60%)
mTICI ≥2b, *n* (%)	51 (64%)	16 (47%)	8 (40%)	6 (46%)	3 (37%)	2 (40%)
mTICI ≥2c, *n* (%)	43 (54%)	12 (35%)	5 (25%)	3 (23%)	1 (12%)	1 (20%)
mTICI 3, *n* (%)	34 (43%)	7 (21%)	2 (10%)	2 (15%)	0 (0%)	0 (0%)

*mTICI, modified thrombolysis in cerebral infarction*.

Advancing the SR was rated “very easy” in 39%, “easy” in 47%, and “neutral” in 14% of our cases, while none of the procedures was rated as “hard.” Target placement of the SR was achieved in all cases. Withdrawing the SR toward the aspiration-catheter to reach the wedge position or retrieve it toward the BGC was rated as “very easy” in 51%, “easy” in 42%, and “neutral” in 7%. The wedge position could be sustained in 91% of the procedures.

## Discussion

Early and complete arterial recanalization is the most important factor in achieving favorable clinical outcome after ischemic stroke due to LVO (10, 11). Mechanical thrombectomy is the standard of care for LVO and gaining momentum for medium vessel occlusions as well, as multiple randomized trials have demonstrated improved patient outcomes in the interventional arm (1, 12). In cases of tortuous proximal vessels or distal target lesions, pushing the SR through the microcatheter and retrieving the SR within the aspiration-catheter can be challenging. The design of the new Trevo NXT is supposed to help in these situations, due to the hydrophilic coating of the pusher wire. In this retrospective multicenter study, we evaluated the initial results with this new device. One of our main findings was that the rate of first-pass complete or near-complete reperfusion (mTICI ≥2c) was achieved in 54% of cases, which compares favorably to previously published data of the predecessor retriever (Table 4) (13, 14). Final mTICI ≥2c was achieved in 83%, and final complete reperfusion (mTICI 3) in 56% of the cases. First-pass mTICI ≥2c was achieved more often than in the Trevo 2000 Registry, where the rate was 28% (13). Regarding final reperfusion rates, mTICI 3 was documented in 56% of the Trevo 2000 Registry cases, which is identical to the final mTICI 3 rates of our study (56%) (7). Data of other newer-generation SRs were mostly comparable to our results: Ribo et al. reported 63% mTICI ≥2b and 47% mTICI ≥2c first-pass reperfusion using the Neva thrombectomy device, although with a small sample size of only 30 patients (16). Slightly inferior results with 52% first-pass mTICI ≥2b and 40% first-pass mTICI ≥2c were reported in the prospective ARISE II study, where the EmboTrap SR was assessed using a larger sample size (n = 227) (15). Results of the Aperio Hybrid SR were recently published, with 52% mTICI ≥2b and 31% mTICI ≥2c first-pass reperfusion in a sample of 48 patients, which are slightly inferior to our reperfusion rates (22). The Tigertriever, which can be radially adjusted, was recently evaluated in the multicentric Tiger Trial (*n* = 160), yielding similar reperfusion results with first-pass successful reperfusion rates of 58% and final near-complete reperfusion rates of 72% (18). Technical approaches varied within all these studies, limiting the degree to which reperfusion results can be compared. For example, while in this study the primarily used technique was a combined technique (93% of the cases), this approach was used only in a minority of cases in the Trevo 2000 Registry (7).

**Table 4:** Overview of recent studies evaluating stent retrievers.

	**Primary used stent retriever**	**Number of patients**	**First-pass reperfusion rate (%)**		**Final reperfusion rate (%)**	
**mTICI**			**≥2b**	**≥2c**	**≥2b**	**≥2c**
Trevo 2000 Registry (7, 13)	Trevo XP	2,008	-	27.8%	92.8%	56.4%^a^
Yi et al. (14)	Trevo XP	98	40.8%		89.7%	
	Solitaire	102	32%		82.3%	
ARISE II (15)	EmboTrap I and II	227	51.5%	40%	92.5%	76%
Ribo et al. (16)	Neva thrombectomy device	30	63%	47%	93%	63%
Velioglu et al. (17)	CatchView thrombectomy device	53	47.2%	-	84.9%	-
Gupta et al. (18)	Tigertriever	160	57.8%	-	95.7%	71.8%
Kurre et al. (19)	pREset stent retriever	271	-	-	84.9%	-
ARTESp (20)	pREset stent retriever	100	-	-	85.3%	-
Kaschner et al. (21)	Aperio thrombectomy device	97	43.9%	15.1%	85.3%	54.8%
Kaschner et al. (22)	Aperio Hybrid stent retriever	48	52.1%	31.3%	95.8%	60.4%
Pfaff et al. (23)	Solitaire Platinum stent retriever	75	38.6%		92%	
Our study	Trevo NXT ProVue retriever	80	63.75%	53.75%	96.25%	82.5%

a *Refers to mTICI 3 results*.

*mTICI, modified thrombolysis in cerebral infarction*.

Regarding the technical aspects using the Trevo NXT as first-line device, advancing the SR within the microcatheter was described as “easy” in the majority of cases, and target placement of the SR was achieved in all cases. Even in cases of curved proximal vessels or tortuous siphon we were able to push the 4 mm Trevo NXT through a 0.021-inch microcatheter without failing to reach the target position. While treating distal occlusions with the 3 mm Trevo NXT, pushing the device through a 0.017-inch microcatheter was feasible. In our subjective opinion, pushing the new 3 mm device through a 0.017-inch microcatheter was easier compared to previous Trevo generations, although we did not compare the 2 devices in this study. In terms of retrieving the SR into the aspiration-catheter or the BGC, we also received positive feedback from the interventionalists. These findings are consistent with the development of a hydrophilic coated polymer jacket, which was designed to enable a smoother and easier delivery and an improved retraction into the aspiration-catheter. Our experience is that even in tortuous proximal vessels it is much easier to push a rather rigid large-bore aspiration-catheter toward the face of the clot over the new SR after removal of the microcatheter. For physicians using the Solumbra technique (full retrieval of the SR within the aspiration-catheter) as their primary approach, the hydrophilic jacket provides an even smoother retrieval experience compared to older SRs. Nevertheless, we would not recommend this technique to be used as first-line approach, based on the higher occurrence of clot fragmentation and distal emboli (24). A potential disadvantage of the new coating is encountered when pulling the SR toward the tip of the aspiration-catheter for the SAVE maneuver (25): With conventional SRs, there is a point where a wedge position is reached and cannot be lost even with significant pulling power on the SR wire, due to entrapment of the clot between the SR and the aspiration-catheter. Using the new device, we noticed that continuous pull after reaching the wedge position can lead to unintentional withdrawal of the SR within the aspiration-catheter (9% of our cases) resulting in an unintended Solumbra maneuver.

Concerning safety, the Trevo NXT retriever can be regarded as safe. Complication rates were comparable to those of the literature (1). Both cases of SAH were unnoticed during the procedure and could not be clearly attributed to the SR (26).

The main limitation of our study is the retrospective design. Patients were included after initial stroke incident and treatment with mechanical thrombectomy. In addition, angiographic results and complications were rated by the treating physician and not by a core lab, which can lead to heterogenous judgments and potentially influence results. Finally, the Trevo NXT was chosen as the first-line device by the treating physician and not allotted in a randomized setup.

## Conclusion

Based on our initial data, we conclude that the Trevo NXT is an effective and safe tool for mechanical thrombectomy especially when using combined approaches.

## Data Availability Statement

The raw data supporting the conclusions of this article will be made available by the authors, without undue reservation.

## Ethics Statement

Ethical review and approval was not required for the study on human participants in accordance with the local legislation and institutional requirements. Written informed consent for participation was not required for this study in accordance with the national legislation and the institutional requirements.

## Author Contributions

AB, PS, IT, KB, and JO designed the data collection sheets and performed the analysis. MMö, CW, CP-G, MMo, FC, NL, ON, and MW contributed the data and helped to evaluate the data. ME and M-NP wrote the manuscript. All authors reviewed the manuscript critically, gave final approval of the submitted version, and agreed to be accountable for all aspects of the work in ensuring that questions related to the accuracy or integrity of any part of the work are appropriately investigated and resolved.

## Conflict of Interest

The authors declare that the research was conducted in the absence of any commercial or financial relationships that could be construed as a potential conflict of interest.
